# *FADS* genotypes shape plasma polyunsaturated fatty acids independently of dietary intake in breast cancer survivors on aromatase inhibitors

**DOI:** 10.3389/fnut.2026.1850476

**Published:** 2026-07-02

**Authors:** Biljana Pokimica, Vesna Vucic, Snjezana Petrovic, Simonida Bobic, Igor Djurisic, Andjelija Petrovic, Maja Milosevic

**Affiliations:** 1 Group for Nutritional Biochemistry and Dietology, Institute for Medical Research, National Institute of the Republic of Serbia, University of Belgrade, Belgrade, Serbia; 2 Centre of Research Excellence in Nutrition and Metabolism, Institute for Medical Research, National Institute of the Republic of Serbia, University of Belgrade, Belgrade, Serbia; 3 Clinic of Medical Oncology, Institute of Oncology and Radiology of Serbia, Belgrade, Serbia; 4 Department of Digestive Surgery, Institute of Oncology and Radiology of Serbia, Belgrade, Serbia

**Keywords:** arachidonic acid, breast cancer survivors, *FADS*, polyunsaturated fatty acids, single nucleotide polymorphism

## Abstract

**Introduction:**

Alterations in lipid metabolism are common in breast cancer (BRC) survivors on aromatase inhibitor therapy, and may increase the risk of cardiovascular complications. Long-chain n-3 and n-6 plasma fatty acids (PUFA) are substantial components of complex lipids, with important structural and regulatory functions. Their circulating levels are determined by both dietary intake and endogenous metabolism, of which the latter is influenced by polymorphisms in fatty acid desaturase (*FADS*) genes. This study aimed to assess the association of *FADS* polymorphisms and plasma n-3 and n-6 PUFA levels in BRC survivors on AI therapy.

**Methods:**

Fatty acid status was determined by gas chromatography, and *FADS* polymorphisms (rs174537, rs174576 and rs174602) were genotyped using real-time PCR. Dietary intake of n-3 and n-6 fatty acids was evaluated via three 24-h dietary recalls. Data were analyzed using a multiple linear regression model.

**Results:**

All three polymorphisms were significantly associated with plasma arachidonic acid levels. Minor alleles of rs174537 (TT), rs174576 (AA) and rs174602 (CC) were associated with lower plasma arachidonic acid proportions. Moreover, positive association of rs174537 and rs174576 minor alleles with higher levels of linoleic acid, precursor of arachidonic acid and decreased estimated activities of delta-5 and delta-6 desaturases were observed, pointing out a lower conversion rate along the n-6 pathway. Lower levels of n-3 docosapentaenoic acid were associated with minor allele carriers of rs174576, while no significant differences were found for other n-3 PUFA. Dietary intake of n-3 and n-6 fatty acids did not differ significantly across the three genotypes, except for EPA for the rs174602 groups.

**Discussion:**

These findings demonstrate that *FADS* genotypes significantly influence plasma PUFA profiles independently of dietary intake in BRC survivors receiving AI therapy. This supports the potential relevance of considering genetic variability in future approaches aimed at optimizing PUFA balance and reducing CVD risk in this vulnerable population.

## 1 Introduction

Breast cancer (BRC) is the most common malignancy in women worldwide (1). The predominant subtype is estrogen receptor-positive (ER+), which is commonly treated with aromatase inhibitors (AI), resulting in profound estrogen depletion (2). Estrogen influences cardiovascular health by regulating various metabolic processes, including lipid metabolism (3). Even healthy postmenopausal women exhibit unfavorably altered circulating lipid profiles (2).

Evidence suggests that estrogen also influences the activity of desaturases, enzymes encoded by fatty acid desaturase 1 (*FADS1*) and 2 (*FADS2*) genes, which are required for the conversion of short to long-chain polyunsaturated fatty acids (PUFA) (4, 5). These enzymes regulate the conversion of the essential dietary PUFA, namely n-6 linoleic acid (LA) to arachidonic acid (AA), and n-3 *α*-linolenic acid (ALA) to n-3 eicosapentaenoic acid (EPA) and docosahexaenoic acid (DHA) (6). Polymorphisms in *FADS* genes influence the efficiency of this conversion, thereby affecting circulating PUFA profiles (7, 8). Long-chain PUFA, particularly AA and EPA, have crucial structural and regulatory functions and, through their bioactive metabolites, play key roles in inflammatory and thrombotic processes (7, 8). The balance between AA and EPA, often expressed as the EPA/AA ratio, has been associated with cardiovascular disease (CVD) risk (9, 10). In addition, genetic variation within the *FADS* cluster has also been linked to cardiovascular conditions (ischemic stroke, aortic stenosis risk, and venous thromboembolism) based on a Mendelian randomization study (11, 12).

In postmenopausal women, metabolic syndrome has been associated with reduced levels of cardioprotective fatty acids in red blood cells, while *FADS1* and *FADS2* polymorphisms contribute to unfavorable fatty acid profiles (13). Although *FADS* polymorphisms have been widely studied, further population-specific research is needed due to sex- and ethnicity-related differences in their effects on desaturase indices (14). Considering the elevated cardiometabolic risk observed in breast cancer survivors on AI therapy, plasma fatty acid profiles and their determinants may provide important insights into their potential risk for vascular diseases. Although some *FADS* polymorphisms have been studied in healthy Serbian individuals (15, 16), data for BRC survivors remain lacking, including variants rs174537 and rs174602 in this population.

Therefore, the aim of our study was to investigate plasma fatty acid status in women undergoing AI therapy, and to examine their association with *FADS* gene polymorphisms (rs174537, rs174576, and rs174602), with potential implications for personalized dietary strategies in BRC survivors.

## 2 Materials and methods

### 2.1 Study population

Breast cancer survivors receiving AI therapy for 6–30 months were recruited between December 2023 and April 2025 from the University Hospital Medical Center Bežanijska Kosa and the Institute for Oncology and Radiology of Serbia, Belgrade. Eligible participants were women aged 45–70 years with histologically confirmed stage IB–IIIA, ER+/PR+, HER2-negative breast cancer. Individuals with other malignancies, serious chronic illnesses, metastatic disease, or active infections were excluded. Informed consent was obtained from all participants prior to their inclusion in the study. This study was conducted in accordance with ethical standards and was approved by the Medical Ethics Committee of the University Hospital Medical Center Bežanijska Kosa, Faculty of Medicine, University of Belgrade (No. 6041/1); the Ethics Committee of the Institute for Medical Research, University of Belgrade (No. EO606/23); and the Ethics Committee of the Institute for Oncology and Radiology (No. O1-1/2024/1443).

### 2.2 Assessment of dietary intake

Dietary intake was evaluated using repeated 24-h recalls, including two weekdays and one weekend day, based on participants’ retrospective self-reports. Data were collected through comprehensive, structured multiple-pass interviews conducted by trained research staff, providing a detailed picture of habitual dietary patterns. Participants recalled all foods and beverages consumed over the preceding 24 h, starting with the first item after waking, and estimated portion sizes using common household measures such as pieces, slices, cups, and spoons. Food quantities were further refined using kitchen utensils and packaging information. Daily energy and nutrient intakes, including macro- and micronutrients, were calculated using the Slovenian Food Composition Database, selected due to its comprehensive nutrient and fatty acid composition data and its frequent use in regional nutritional studies where national databases are limited, supplemented with customized recipes and brand-specific information for traditional and locally specific Serbian foods (17).

### 2.3 Blood samples collection

Blood samples were collected in the morning between 8:00 and 9:00 a.m. after an overnight fast of 8–12 h. Samples were drawn into both anticoagulant-free tubes and tubes containing ethylenediaminetetraacetic acid (EDTA).

Plasma was isolated from EDTA-treated blood by centrifugation at 2500 × g for 10 min and stored at −80 °C until further analysis. The buffy coat was collected from EDTA tubes following the initial centrifugation and subsequently used for DNA extraction on the same day.

### 2.4 Fatty acid analysis

Fatty acids (FAs) were extracted from total plasma lipids following previously established protocols (18, 19). The extracted FAs were directly transesterified using 3 N HCl in methanol at 85 °C for 60 min. The resulting fatty acid methyl esters (FAMEs) were analyzed using a SHIMADZU 2014 gas chromatograph equipped with a RESTEK Rtx-2330 capillary column. The oven temperature was programmed to increase from 140 °C to 210 °C at a rate of 3 °C/min. Individual FAs were identified by comparing their retention times with those of commercial FAME standards (PUFA-2; Supelco, Inc., Bellefonte, PA, USA). The FA composition is expressed as a percentage of total identified FA. Delta 5 desaturase activity was estimated as ratio AA/DGLA, and delta-6 desaturase activity was estimated as the ratio GLA/LA.

### 2.5 Genotyping

Polymorphisms were selected based on literature data, based on their position and functional annotation (20).^1^ Genomic DNA was extracted from buffy coat using a commercial kit (PureLink Genomic DNA Mini Kit, Invitrogen) following the manufacturer’s instructions. Genotyping of polymorphisms (rs174537, rs174576, and rs174602) was performed using PCR-based TaqMan assays. DNA samples were incubated with Genotyping Master Mix (Applied Biosystems) and specific assays according to the manufacturer’s protocol.

### 2.6 Statistical analysis

Deviation from the Hardy–Weinberg equilibrium was assessed using the chi-square test. Plasma fatty acid levels were stratified by genotype and compared using linear regression adjusted for BMI, dietary intake of appropriate fatty acids (precursors and/or long-chain fatty acids) and duration of AI therapy. Dietary fatty acid intakes were also compared between genotypes using Kruskal Wallis test and Mann Whitney U test. Statistical analyses were performed in SPSS.

## 3 Results

### 3.1 Characteristics of the study population

A total of 72 BRC survivors were included in study, with a mean age of 58.22 ± 8.77 years and a mean BMI of 26.43 ± 4.41 kg/m^2^. Prior to study enrollment, the majority of participants (*n* = 22) had stage IIB tumors, while 17 had stage IB, 17 had IIA disease and 16 had stage IIIA tumors.

### 3.2 Dietary intake of PUFA

Based on three validated 24-h dietary recalls (24HDR), the mean dietary intake of LA was 11.74 ± 6.28 g, while the mean total PUFA intake was 13. 68 ± 7.19 g. The mean intake of ALA, a precursor for long chain EPA and DHA, was 1.75 ± 1.50 g. The mean dietary intake of AA was 0.15 ± 0.10 g, while the intakes of EPA and DHA were 0.12 ± 0.16 g and 0.30 ± 0.35 g, respectively. No significant differences in dietary intake was observed among the three genotypes of each polymorphism, except for dietary intake of EPA, which differed significantly across rs174602 genotypes (*p* = 0.035).

### 3.3 *FADS* allele and genotype distributions

Genotype distributions for all three analyzed SNPs were consistent with Hardy–Weinberg equilibrium (*p* > 0.05). Minor allele frequencies for rs174537 and rs174576 were comparable to those reported by 1,000 genomes (Table 1).

**Table 1:** Characteristics of investigated polymorphisms.

SNP	Chromosome position	Functional annotation	Major/minor allele	Minor allele frequency	1,000 genomes minor allele frequency	HWE *p*
rs174537	11:61785208 11:61552680	Intron variant	G/T	0.25	0.30	0.12
rs174576	11:61836038 11:61603510	Intron variant	C/A	0.30	0.36	0.81
rs174602	11:61856942 11:61624414	Intron variant	T/C	0.25	0.42	0.12

The positions of the gene variants and minor allele frequencies for the 1,000 genomes were retrieved from the National Center for Biotechnology Information (NCBI) database (https://www.ncbi.nlm.nih.gov/). HWE, Hardy–Weinberg equilibrium.

### 3.4 Plasma fatty acid profile association to *FADS* genotype

Significant negative associations were observed between plasma AA levels and minor alleles of rs174537 (*β* = −0.49, *p* < 0.001), rs174576 (*β* = −0.55, p < 0.001), and rs174602 (*β* = −0.27, *p* = 0.03) (Tables 2–4). Moreover, for both rs174537 and rs174576 genotypes level of LA was higher, with an increasing number of minor alleles (*β* = 0.32, *p* = 0.008 and *β* = 0.33, *p* = 0.006), indicating a moderate positive effect for the additional minor allele (Tables 2, 3). For DGLA (20:3n-6) there was a positive, but non-significant trend for rs174537 minor alleles (*β* = 0.23, *p* = 0.057) (Table 2). Minor alleles of rs174576 were associated with a slightly lower EPA (*β* = −0.25, *p* = 0.05) and docosapentaenoic acid (DPA) levels (*β* = −0.29, *p* = 0.024) (Table 3).

**Table 2:** Polyunsaturated fatty acid status by genotypes of rs174537 polymorphism.

Gene variant	rs174537			*p*-value (genotype)
	GG	GT	TT	
Linoleic acid (%)	23.94 ± 3.72	25.25 ± 3.22	27.17 ± 2.43	**0.008**
*γ*-linolenic acid (%)	0.21 ± 0.10	0.18 ± 0.09	0.18 ± 0.07	0.253
Dihomo-*γ*-linolenic acid (%)	2.33 ± 0.58	2.67 ± 0.83	2.71 ± 0.41	0.057
Arachidonic acid (%)	12.10 ± 2.51	10.71 ± 2.18	7.47 ± 2.67	**<0.001**
α-linolenic acid (%)	0.16 ± 0.08	0.16 ± 0.08	0.20 ± 0.12	0.391
Eicosapentaenoic acid (%)	0.42 ± 038	0.33 ± 0.22	0.25 ± 0.15	0.118
Docosapentaenoic acid (%)	0.39 ± 0.16	0.36 ± 0.12	0.32 ± 0.14	0.113
Docosahexaenoic acid (%)	2.06 ± 0.76	1.92 ± 0.75	1.86 ± 1.19	0.370
Delta 5 desaturase	5.47 ± 1.61	4.33 ± 1.31	2.83 ± 1.31	**<0.001**
Delta 6 desaturase	0.01 ± 0.01	0.01 ± 0.01	0.01 ± 0.00	**0.037**

Statistically significant *p*-values (*p* < 0.05) are presented in bold.

**Table 3:** Polyunsaturated fatty acid status by genotypes of rs174576 polymorphism.

Gene variant	rs174537			*p*-value (genotype)
	GG	GT	TT	
Linoleic acid (%)	23.79 ± 3.78	24.99 ± 3.16	27.97 ± 1.61	**0.006**
γ-linolenic acid (%)	0.22 ± 0.10	0.18 ± 0.09	0.18 ± 0.05	0.214
Dihomo-γ-linolenic acid (%)	2.39 ± 0.58	2.48 ± 0.76	2.89 ± 0.47	0.193
Arachidonic acid (%)	12.48 ± 2.37	10.68 ± 2.24	6.74 ± 1.59	**<0.001**
α-linolenic acid (%)	0.15 ± 0.07	0.16 ± 0.09	0.22 ± 0.12	0.142
Eicosapentaenoic acid (%)	0.44 ± 0.40	0.33 ± 0.22	0.20 ± 0.08	**0.050**
Docosapentaenoic acid (%)	0.41 ± 0.17	0.35 ± 0.11	0.31 ± 0.15	**0.024**
Docosahexaenoic acid (%)	2.12 ± 0.77	1.93 ± 0.72	1.63 ± 1.16	0.105
Delta 5 desaturase	5.49 ± 1.57	4.65 ± 1.47	2.33 ± 0.47	**<0.001**
Delta 6 desaturase	0.01 ± 0.01	0.01 ± 0.00	0.01 ± 0.00	**0.017**

Statistically significant *p*-values (*p* < 0.05) are presented in bold.

**Table 4:** Polyunsaturated fatty acid status by genotypes of rs174602 polymorphism.

Gene variant	rs174602			*p*-value (genotype)
	TT	TC	CC	
Linoleic acid (%)	24.15 ± 3.78	25.30 ± 3.24	25.72 ± 2.71	0.160
γ-linolenic acid (%)	0.20 ± 0.10	0.18 ± 0.08	0.20 ± 0.12	0.719
Dihomo-γ-linolenic acid (%)	2.44 ± 0.66	2.46 ± 0.63	2.69 ± 0.84	0.424
Arachidonic acid (%)	11.91 ± 2.79	10.27 ± 2.64	10.02 ± 1.47	**0.03**
α-linolenic acid (%)	0.17 ± 0.08	0.13 ± 0.05	0.23 ± 0.14	0.580
Eicosapentaenoic acid (%)	0.43 ± 0.38	0.24 ± 0.10	0.46 ± 0.26	0.368
Docosapentaenoic acid (%)	0.40 ± 0.17	0.31 ± 0.05	0.39 ± 0.15	0.155
Docosahexaenoic acid (%)	2.07 ± 0.78	1.75 ± 0.55	2.33 ± 1.28	0.775
Delta 5 desaturase	5.19 ± 1.72	4.49 ± 1.65	4.01 ± 1.16	**0.043**
Delta 6 desaturase	0.01 ± 0.01	0.01 ± 0.00	0.01 ± 0.00	0.477

Statistically significant *p*-values (*p* < 0.05) are presented in bold.

## 4 Discussion

We evaluated plasma fatty acids in BRC survivors receiving AI therapy, given their potential adverse impact on lipid metabolism, with a focus on *FADS* genotypes as a key source of interindividual variability. Among our patients, no significant associations were observed between the three *FADS* genotypes and dietary intake of AA or LA, indicating that the observed differences in fatty acid profiles are primarily driven by endogenous metabolism rather than diet. AI therapy induces estrogen depletion, which may alter desaturase activity, thereby contributing to the observed alterations in fatty acid metabolism and potentially increasing cardiometabolic risk in this population.

Our results demonstrated significant differences in plasma AA levels across genotypes of all three investigated polymorphisms (rs174537, rs174576, and rs174602) in BRC survivors. Consistent with this interpretation, minor alleles of rs174537 and rs174576 were additionally associated with higher LA levels, and lower estimated delta-5 and delta-6 desaturase activities, indicating reduced conversion efficiency along the n-6 metabolic pathway.

These findings are in line with previous studies showing that *FADS* variants modulate AA levels across different populations, although the results and associations depend on the investigated polymorphisms (8, 15, 21). However, evidence in BRC survivors, particularly those receiving AI therapy, remains limited (22). Our results extend existing evidence by demonstrating similar genetic effects in this specific clinical context.

The observed associations between PUFA levels and *FADS* polymorphisms may be particularly significant for BRC patients on AI due to intensive estrogen depletion. Estrogens are well-known regulators of PUFA biosynthesis. Experimental studies suggest that estradiol regulates *FADS2* transcription through both estrogen receptor-mediated mechanisms and activation of the ERK1/2-MAPK–PPARα pathway, while progesterone may additionally enhance *FADS* expression through epigenetic regulation (23). In healthy postmenopausal women, residual estrogen production persists due to peripheral aromatization in adipose tissue, but in BRC patients, AI therapy further suppresses this synthesis, leading to almost complete estrogen deprivation (24). Thus, AI-induced estrogen depletion might synergize with genotype and affect the PUFA levels to a greater extent compared with healthy postmenopausal women.

These findings may have implications for personalized dietary approaches. Individuals carrying major alleles of rs174537, rs174576, and rs174602, who appear to have a greater capacity for AA synthesis, may potentially benefit from monitoring dietary intake of LA and increasing n-3 PUFA intake, given the shared enzymatic pathways involved in n-3 and n-6 metabolism (4). In addition, maintaining an appropriate balance between n-3 and n-6 fatty acids remains an important consideration for overall metabolic and cardiovascular health. No significant differences in n-3 PUFA dietary intake were observed across genotypes, except for EPA intake in rs174602, which was the highest in major allele carriers. However, this difference was not reflected in plasma fatty acid status, suggesting that dietary intake may have been sufficient to maintain relatively stable plasma fatty acid levels across genotype-stratified groups.

Previous studies have reported an association between certain *FADS* polymorphisms, including rs174537, and circulating EPA and DHA levels (25). In the present study, no significant association was observed for EPA, whereas carriers of the minor allele of rs174576 exhibited lower plasma DPA levels. This occurred despite comparable dietary intake of ALA and EPA. Therefore, our results may indicate reduced conversion efficiency within the n-3 conversion pathway among minor allele carriers. Further studies with larger sample sizes are needed to confirm this finding.

## 5 Limitations

The relatively small number of patients represents a potential limitation. However, this sample size is comparable to other studies investigating *FADS* polymorphism in both healthy (15, 16) and diseased populations (26). Further, although three 24-h dietary recalls are a validated and commonly accepted method in nutritional studies, it should be noted that it is still susceptible to bias.

## 6 Conclusion

This study provides novel evidence on the role of *FADS* polymorphisms (rs174537, rs174576, and rs174602) in shaping plasma PUFA profiles independently of dietary intake in BRC survivors undergoing AI therapy. These findings contribute to a better understanding of interindividual variability in fatty acid metabolism in a population characterized by clinically relevant metabolic alterations, particularly in lipid metabolism.

Overall, our findings highlight the significant influence of the investigated polymorphisms (rs174537, rs174576, and rs174602) in influencing plasma PUFA profiles of BRC survivors. Therefore, these results provide a preliminary basis for future interventional trials incorporating nutrigenetic information into personalized nutritional approaches that may help reduce cardiometabolic risk in BRC survivors.

## Data Availability

The original contributions presented in this study are included in the article. Further inquiries can be directed to the corresponding author.

## Footnotes

1
https://regulomedb.org/regulome-search
